# Do Brazilian regulatory measures promote sustainable and healthy eating in the school food environment?

**DOI:** 10.1186/s12889-023-17111-7

**Published:** 2023-11-06

**Authors:** Luana Lara Rocha, Nayhanne Gomes Cordeiro, Mariana Zogbi Jardim, Aline Yukari Kurihayashi, Patrícia Chaves Gentil, Giorgia Castilho Russo, Larissa Loures Mendes

**Affiliations:** 1 Universidade Federal de Minas Gerais, Belo Horizonte, MG Brazil; 2 Universidade Federal do Rio de Janeiro, Rio de Janeiro, RJ Brazil; 3 Brazilian Institute for Consumer Protection-IDEC, Brazil, Brazil; 4 Departamento de Nutrição, Universidade Federal de Minas Gerais, Belo Horizonte, MG Brazil; 5 Av. Alfredo Balena 190 Santa Efigênia, Belo Horizonte, 30130-100 Brazil

**Keywords:** Schools, Legislation, Food Environment, Score

## Abstract

**Background:**

Regulatory measures regarding food in the school environment aim to promote a healthier food environment in public and private schools. In Brazil, implementing regulations in the school food environment does not occur the same way across states and cities, and no national regulation covers public and private schools. The present study aims to analyze regulatory measures for school food environments in Brazilian states and cities and develop a score to evaluate them.

**Methods:**

A systematic search of the regulatory measures in force and implemented until 2021 was conducted. The score was developed based on the Model Law Project prepared by the Brazilian Institute for Consumer Protection. It considered food and nutrition education actions, restrictions on the sale and distribution of food, a ban or restriction on food advertising and marketing, and points of excellence. These points included regulations that addressed the importance of supervision and social control, laws regulated by decree, the mention of a ban on ultra-processed foods, and whether the regulatory measures covered public and private schools.

**Results:**

Sixty-five cities and states regulatory measures in force were found to be evaluated jointly by a federal entity (n = 43). Among the federal entities evaluated, only 13.95% fulfilled the function of promoting sustainable and healthy eating (8–12 points).

**Conclusions:**

Brazilian children and adolescents are exposed to a school food environment with regulations that partially fulfill the function of promoting an adequate, healthy, and sustainable diet. In this sense, it is necessary to improve regulatory measures or to encourage states and cities to develop effective legal provisions that are in line with the food guide for the Brazilian population and with the perspective of a healthy school food environment for the effective promotion of adequate, healthy and sustainable and healthy food in schools.

**Supplementary Information:**

The online version contains supplementary material available at 10.1186/s12889-023-17111-7.

## Background

The school food environment is defined by the Food and Agriculture Organization (FAO) as the space, infrastructure, and conditions within and around schools in which food is available and can be obtained, purchased, and consumed, such as canteens, cafeterias, self-service machines, street vendors and any facility where food is sold. It also includes all available information, promotion (marketing, advertisements, brands, food labels, packaging), and food products’ pricing [1]. The physical presence and proximity to these establishments and any means that allow access to food within and around schools affect the dietary patterns of these children and adolescents [2, 3]. Thus, studying the school food environment is considered a strategic element in understanding the influence of this environment on the food choices and nutritional status of children and adolescents [1, 4, 5].

Unhealthy foods in and around schools follow a global pattern. A study in two cities in Mexico showed that schools were surrounded by establishments selling unhealthy foods and drinks. Furthermore, advertisements for these products were located outside the establishments, where children would be most exposed [6]. In Kenya, a high presence of street vendors has been noted in the vicinity of schools, offering a variety of unhealthy foods [7]. Similar results were identified in India, where unhealthy foods were widely available within and in educational institutions [8]. In New Zealand, an evaluation of school menus revealed that most schools do not comply with Ministry of Health recommendations, providing children with unhealthy foods [9].

In Brazil, according to the 2022 School Census by the National Institute of Educational Studies and Research Anísio Teixeira (Inep), public schools represent 82.9% of total school enrollment, while private schools represent 17.1% [10]. These spaces play an essential role in food consumption, given that children and adolescents consume 30 to 50% of their daily food intake within the schools [11]. According to the National School Health Survey (Pesquisa Nacional de Saúde do Escolar, PeNSE) carried out in Brazil in 2019, 88.0% of private schools and 31.4% of the public schools have canteens that sell food, and 55.6% of the school canteens evaluated sell soft drinks [12]. In addition, 97.3% of Brazilian students aged 13 to 17 consumed at least one ultra-processed food the day before PeNSE data collection [12].

Therefore, we highlight the importance of implementing regulatory measures for the school food environment. Such measures should include all the multiple dimensions and aim to promote a healthy environment in public and private schools by reducing the availability and access to ultra-processed foods and beverages and increasing the supply of fresh and minimally processed foods [13–16].

It is noted that, in Brazil, regulations aiming to promote a healthy school food environment occur in different ways across the states and cities, and no national regulation exists and covers public and private schools. Furthermore, in locations with legal provisions regulating this environment, there is no compliance monitoring or studies that assess the effectiveness of these measures and the real impacts on children and adolescent’s health [17].

However, even if it doesn’t have the force of Law, there are many governmental and non-governmental efforts to regulate commercial and advertising practices in school environments. For example, Interministerial Ordinance Number 1,010 [18] establishes the national guidelines for Promoting Healthy Eating in Schools in the public and private sectors. The National Education Development Fund (Fundo Nacional de Desenvolvimento da Educação, FNDE) released the Technical Note Number 2,974,175 of 2022 [19], which reinforces that advertising, publicity, or promotion through sponsorship of school activities, including extracurricular activities, publicizing special presentations and distributing gifts, prizes or bonuses for unhealthy foods, preparations or beverages is prohibited in public schools. In the non-governmental sector, the Brazilian Institute for Consumer Protection (Instituto Brasileiro de Defesa do Consumidor, Idec) [20] brought together various experts in the field of food and health and proposed a Model Law Project, which aims to formulate and analyze regulatory standards to promote public and private schools that encourage a school environment that supports adequate and healthy food.

Besides these efforts, it is essential to mention that any regulatory measures focusing on the school environment will encounter different circumstances, especially in public schools compared to private schools. One of the main differences in the food environment between these types of schools is the presence of the National School Feeding Program (Programa Nacional de Alimentação Escolar, PNAE) [21, 22]in the public sector. The PNAE can promote the regulation of the supply and acquisition of food with public resources and other strategies that foster healthy eating in these environments.

In addition to the existence of regulatory measures, assessing whether they promote a healthy school food environment is necessary. In the meantime, there is no tool for evaluating regulatory measures in Brazil, making it difficult to understand and monitor the scenario. It is worth mentioning the existence of the *Classification of Laws Associated with Schools Students* (CLASS), an international tool for evaluating regulatory measures aimed at schools. However, it is not possible to adapt this tool to assess Brazilian regulations since it includes particular characteristics of American schools, which are different from the Brazilian reality [23].

Thus, due to the absence of a suitable tool to assess the current regulations in Brazil regarding the schools’ food environment and the significance of regulating food and beverage trade in these spaces, this study aims to analyze regulatory measures for school food environments in Brazilian states and cities and apply the score to evaluate them.

## Methods

### Data collection

Data collection included the search for regulations, carried out in duplicate by previously trained researchers, who consulted official websites of the city and state governments of the Brazilian capitals. The regulatory measures implemented until 2021 were verified through (a) consultation with the websites of the official government agencies, (b) review of the gray literature, and, when necessary, (c) contact by email. The search was carried out with the help of the Legislative Process Support System (SAPL). When necessary, the Google Chrome internet browser was used [24], without a date filter and using the terms in Portuguese “cantinas”, “alimentação escolar”, “legislação”, “regulamentação”, and “escolas”. The regulations selected were only those that dealt with some aspect of the school food environment.

Two researchers (N.G.C. and M.Z.J) read the text of the regulations independently, and a third researcher (L.L.M) was consulted to discuss doubts. After reading and selecting the legal provisions, the regulatory measures were evaluated. The present study understands that the legal provisions differ by strength and function. The law is the competence of the legislative branch, which represents the population, hosts debates of interest to society, and allows the creation, extinguishing, or modification of rights and obligations. Thus, the law has greater strength from a hierarchical and democratic point of view. In turn, decrees are issued by the executive branch and may regulate the law, provide details for its execution, and have less force than the law. Resolutions are internal legislative acts that aim to explain regulations and internal rules, having less force than decrees. Ordinances are administrative acts that aim to discipline the public administration and have the least strength from a hierarchical point of view. Finally, normative instructions are administrative acts to complement Laws, Decrees, and Ordinances without transposing them, modifying their text, or making innovations [25].

### Assessment of regulatory measures

The legal devices received an identification number and data extraction based on the data collection. The characterization of regulatory measures was based on information collection: macro-regions (North, Northeast, Midwest, Southeast, and South), administrative regions (state), location (city), year of publication, type of legal provision (resolution, normative instruction, ordinance, law or decree), scope (public and/or private school) and current situation (in force or repealed). Repealed regulatory measures (n = 4) were not analyzed in this study.

### The score

The score was proposed based on the Model Law Project. In 2018, the Idec produced the document “Healthy Eating in Schools: Guide for Municipalities”, aimed at public managers and technical teams in the health and education areas to promote healthy school food environments as one of the actions to confront childhood and adolescent obesity. One of the measures recommended in the Guide is the development of effective legal provisions to assist school managers in promoting healthy school eating environments. As a tool to assist municipal managers in developing these legal provisions, a Model Law Project was presented, with the necessary information for developing effective measures to promote a healthy school food environment [26].

The Model Law Project was revised in 2022 [20, 26] and is based on seven school food environment domains. These domains were used to build the score to evaluate the existing and current regulatory measures for the school food environment in Brazilian states and cities. The domains comprise: (1) Food and Nutritional Education; (2) Distribution and Marketing of Food; (3) Marketing Communication; (4) Supervision of the Implementation of the Regulatory Measure; (5) Scope of the Regulatory Measure; (6) The force of the Regulatory Measure; (7) Mention of Ultra-processed Foods. The last one is supported by the dietary guidelines issued by the Dietary Guide for the Brazilian Population of 2014, which is based on the NOVA classification that foods are categorized according to the extent and degree of processing as unprocessed or minimally processed foods, processed foods, processed culinary ingredients and ultra-processed foods [27].

Based on the Model Law Project [20], the score was proposed to evaluate the regulatory measures aimed at the school food environment. Based on a sum of points, the score aims to evaluate the regulatory measures in force in Brazilian states and cities. The classification, based on the score, allowed the measures to be grouped into three categories according to the number of points received:

- 0 to 3 points: Regulatory measures exist and need to be improved to fulfill their function of promoting sustainable, adequate and healthy eating in the school food environment;
- 4 to 7 points: Regulatory measures partially fulfill their function of promoting sustainable, adequate and healthy eating in the school food environment;
- 8 to 12 points: Regulatory measures fulfill their function of promoting sustainable, adequate and healthy eating in the school food environment.

For the evaluation of regulatory measures, they were grouped by federal entity (Supplementary Material 1). When a state and city had more than one regulatory measure (two laws and one decree, for example), this set was evaluated and considered at the time of score application. A complete reading of the measures found by the federal entity was carried out. If any measure of this set of federal entities had one of the items evaluated, the entire group received the score. This joint evaluation is justified by the complementary nature of the regulatory measures in force in their respective federal entity, categorized between states and cities. The scoring guide can be found in Table 1, and the manual for its application is available in Supplementary Materials.

**Table 1:** Scoring guide for evaluating the regulatory measures regarding the school food environment in the Brazilian federal entities

Scoring	Food and Nutritional Education.
**0**	It is not mentioned in the regulatory measure
**1**	It is mentioned in the regulatory measure, with no provisions for its development
**2**	It is mentioned in the regulatory measure and provides for its development
**Scoring**	**Distribution and Marketing of Food**
**0**	No type of regulation of the distribution and marketing of food in the school environment is mentioned in the regulatory measure
**1**	The regulation of the distribution and marketing of food in the school environment is mentioned in the regulatory measure, without distinguishing which foods are prohibited or allowed
**2**	The regulation of the distribution and marketing of food in the school environment is mentioned in the regulatory measure, distinguishing which foods are prohibited or allowed
**Scoring**	**Marketing Communication**
**0**	There is no mention of any type of regulation of marketing communication in the school environment in the regulatory measure
**1**	Marketing communication in the school environment is prohibited by the regulatory measure
**2**	Marketing Communication of foods not allowed in the school environment is prohibited by the regulatory measure, and describes the prohibited resources
**Scoring**	**Points of Excellence**
**1**	There is a regulatory measure that provides for supervision and social control (by health surveillance, consumer protection agencies, parent-teacher association or education agency)
**1**	There is a regulatory measure addressing private schools
**1**	There is a regulatory measure that is a law
**2**	There is a regulatory measure that is a law and is regulated by a decree
**1**	There is a regulatory measure that prohibits ultra-processed foods
**Final Scoring**:	**Final Classification**
**0–3**	Regulatory measures exist and need to be improved to fulfill their function of promoting sustainable, adequate and healthy eating in the school food environment
**4–7**	Regulatory measures partially fulfill their function of promoting sustainable, adequate and healthy eating in the school food environment
**8–12**	Regulatory measures fulfill their function of promoting sustainable, adequate and healthy eating in the school food environment

### Statistical analysis

Data tabulation was performed using Microsoft Excel version 2010. Descriptive analyses included calculating absolute and relative frequencies, measures of central tendency, and dispersion. The Stata software version 14.0 was used to conduct the descriptive analyses.

## Results

The search showed 65 municipal and state regulatory measures (Supplementary Material 1) in force with the full text available for evaluation. The year of publication for the regulatory measures evaluated ranged from 2001 to 2021, with a median of 2011. For the states, the year of publication ranged from 2001 to 2021, with a median of 2013. For the cities, the year of publication ranged from 2001 to 2019, with a median of 2009. When characterizing the regulatory measures published between 2001 and 2011 and between 2012 and 2021, 37 (56.92%) were published in the first 10 years (2001–2011), and 28 (43.09%) were published in the last 10 years (2012–2021).

The legal provisions addressed in the present study were law (78.46%, n = 51), ordinance (7.69%, n = 05), resolution (4.62%, n = 03), decree (7.69%, n = 05), and normative instruction (1.54%, n = 1). Of these provisions, 29.23% are from the southeast region and 23.08% from the northeast region, 60% belong to Brazilian states (of these states, 63.16% have more than one regulatory measure), 75.38% address public and private schools, 56.92% do not mention foods that can be sold in schools, 75.38% mention foods that cannot be sold in schools and only one regulatory measure mentions the NOVA classification of food (Table 2).

**Table 2:** Description of Brazilian regulatory measures regarding the school food environment (n = 65)

Variables	Absolute frequency	Relative frequency (%)
Region		
*North*	7	10.77
*Northeast*	15	23.08
*South*	12	18.46
*Southeast*	19	29.23
*Midwest*	12	18.46
**Location**		
*State*	39	60.00
*Municipality*	26	40.00
**Scope**		
*Public*	14	21.54
*Private*	2	03.08
*Both public and private*:	49	75.38
**Mentions allowed foods**		
*Yes*	28	43.08
*No*	37	56.92
**Mentions foods not allowed**		
*Yes*	49	75.38
*No*	16	24.62
**Mentions the NOVA classification**		
*Yes*	1	01.54
*No*	64	98.46

Regarding the score evaluation, 43 sets of regulatory measures that obtained a median score of 4 points (minimum = 1 point; maximum = 11 points) were analyzed by the federal entity. Regarding the cities, 23 sets of regulatory measures (26 legal provisions) obtained a median score of 4 points (minimum = 2 points; maximum = 10 points) were evaluated. Regarding the states, 20 regulatory measures (39 legal provisions) obtained a median score of 4 points (minimum = 1 point; maximum = 11 points) were evaluated.

In the Food and Nutrition Education field, 58.14% of the sets of regulatory measures of the federal entities do not mention any incentive and obligation to develop these actions, 44% of which are city and 56% are state (Table 3). Regarding the Distribution and Marketing of Food, 13.95% of the sets of regulatory measures have some restrictions on the sale and distribution of food. However, without specifying which foods are prohibited or allowed (83.33% city and 16.67% state), and 11.63% do not have any type of restriction (20% city and 80% state) (Table 3). For Marketing Communication, 72.09% of the sets of regulatory measures do not mention any restriction or prohibition of advertising and marketing actions in the school food environment, 58.06% of which are city and 41.94% are state (Table 3).

**Table 3:** Evaluation of the scoring of the regulatory measures regarding the school food environment of Brazilian federal entities (n = 43)

Evaluation Domain	Total n	Cities n (%)	States n (%)
*Food and Nutritional Education*			
0	25	11 (44.00)	14 (56.00)
1	12	10 (83.33)	2 (16.67)
2	6	2 (33.33)	4 (66.67)
*Distribution and Marketing of Food*			
0	5	1 (20.00)	4 (80.00)
1	6	5 (83.33)	1 (16.67)
2	32	17 (53.13)	15 (46.87)
*Marketing Communication*			
0	3	18 (58.06)	13 (41.94)
1	0	0 (0.00)	0 (0.00)
2	12	5 (41.67)	7 (58.33)
**Points of Excellence**			
*There is a regulatory measure that provides for supervision and social control*			
0	28	17 (60.71)	11 (39.29)
1	15	6 (40.00)	9 (60.00)
*There is a regulatory measure that addresses private schools*			
0	14	8 (57.14)	6 (42.86)
1	29	15 (51.72)	14 (48.28)
*There is a regulatory measure that is a law*			
0	5	2 (40.00)	3 (60.00)
1	38	21 (55.26)	17 (44.74)
*There is a regulatory measure that is a law and is regulated by a decree*			
0	40	22 (55.00)	18 (45.00)
2	3	1 (33.33)	2 (66.67)
*There is a regulatory measure that prohibits ultra-processed foods*			
0	42	23 (54.76)	19 (45.24)
1	1	0 (0.00)	1 (100.00)
**Final Scoring**			
0–3	13	8 (61.54)	5 (38.46)
4–7	24	11 (45.83)	13 (54.17)
8–12	6	4 (66.67)	2 (33.33)

Note: 23 cities and 20 states

As for the points of excellence, 65.12% of the sets of regulatory measures do not provide for any supervision and social control (60.71% city and 39.29% state), 67.44% of the sets of measures address private schools (51.72% city and 48.28% state), for 88.37% of the sets of measures there are laws (55.26% city and 44.74% state), but only 6.98% are regulated by a decree (33.33% city and 66.67% state). Only one regulatory measure (state) prohibits ultra-processed foods (using the NOVA terminology for the classification of food) in the school food environment (Table 3). In the final scoring, 13.95% of the sets of regulatory measures of the federal entities fulfill their function of promoting sustainable, adequate, and healthy eating in the school food environment, 66.67% of which are city and 33.33% state (Table 3).

The lists of permitted and prohibited foods were not present in all federal entities; only 17 sets of regulatory measures had lists of permitted foods (14 cities and three states), and 30 sets of regulatory measures had lists of prohibited foods (19 cities and 11 states). There were 237 mentions of permitted foods (77 in cities and 160 in states) and 530 mentions of prohibited food (210 in cities and 320 in states). The five most common foods on the list of permitted foods were milk-based drinks, fruit, cakes and bread, natural juice, and vegetables. It is worth noting that milk-based drinks are classified as ultra-processed foods, and cakes and breads as culinary preparations or processed foods, depending on the production method. Furthermore, the five most frequent foods on the list of prohibited foods were sweetened drinks and powdered soft drinks, fried snacks, alcoholic beverages, industrialized cookies and cookies, and popcorn (made from fresh corn kernels).

Regarding the set of regulatory measures related to cities, among the lists of permitted foods, 31.17% were classified as processed culinary ingredients, 27.27% as unprocessed and minimally processed foods, 22.08% had vague claims about the nutrient content of foods, 18.18% were classified as ultra-processed foods and 1.30% as processed foods; and among the list of prohibited foods, 60.95% were classified as ultra-processed foods. Also, 13.81% had vague claims about the nutrient content of foods, 13.33% were classified as processed culinary ingredients, 6.19% as unprocessed and minimally processed foods, 5.24% as non-food products (medicines, tobacco, and alcohol) and 0.48% as processed foods.

Regarding the set of regulatory measures related to the states, among the lists of permitted foods, 33.75% were classified as ultra-processed foods, 30.63% as processed culinary ingredients, 30% as unprocessed and minimally processed foods, 3.75% as processed foods, and 1.87% had vague claims about the nutrient content of the foods. Among the lists of prohibited foods, 53.13% were classified as ultra-processed foods, 29.38% had vague claims about the nutrient content of the foods, 6.87% were non-food products, 6.25% were classified as processed culinary ingredients and 4.37% as unprocessed and minimally processed foods.

## Discussion

The present study evaluated 65 municipal and state regulatory measures in force with full text available for evaluation. They were grouped into 43 sets of measures per federal entity. Of these sets of measures evaluated by the score, 13.95% obtained 8–12 points and were considered a set of legal provisions of a federal entity promoting sustainable, adequate, and healthy eating in the school food environment. However, the others in the set of regulatory measures had lower scores, which means that Brazilian children and adolescents, even with the existing regulations, are still exposed to a susceptible school food environment promoting unhealthy food choices.

It is noteworthy that, in the score for the evaluation of the set of regulatory measures, it is essential to identify which measures do not include all domains that influence the diet of children and adolescents in the school food environment, as well as being the basis for the reformulation of such regulatory measures. In addition, creating a score can support states and cities in building healthier school food environments.

Four domains were considered for the development of the score. In the first domain, which addressed Food and Nutrition Education (FNE) actions, more than half of the sets of regulatory measures evaluated received a score of zero, as they did not mention any incentive or obligation for the development of FNE actions in the school. FNE activities in the school environment are important strategies for integrating food practices with a pedagogical character and topics focused on food and nutrition to build transversal knowledge using active and interactive processes [28, 29]. Thus, with the increase in the prevalence of non-communicable diseases, such as overweight and obesity in children and adolescents, it is essential that regulatory measures include FNE practices and provide subsidies for their execution in a school food environment that promotes health [28–30].

It was also found that most of the sets of regulatory measures restrict the sale and distribution of food. However, 13.95% still do not specify the sale of which foods are permitted or prohibited. The presence of lists of permitted or prohibited foods is inconsistent, with some ultra-processed foods (such as turkey breast, chocolate, and gelatin) being allowed and some unprocessed and minimally processed foods (such as popcorn made from fresh corn kernels) being prohibited. The regulation of the means of Distribution and Marketing of Food in the school food environment is critical, as it is a space in which children and adolescents spend a third of the day and consume about 30 to 50% of the daily food intake during school hours [11, 14], and because of the influence of the food offering on the food choices of children and adolescents [31].

Thus, we highlight the need to encourage and prioritize offering unprocessed and minimally processed foods within the school environment rather than ultra-processed foods. This is guided by the 2014 Food Guide for the Brazilian population and the Food Guide for Brazilian children under two years old [32, 33].

Regarding Marketing Communication on the prohibition or restriction of food advertising and marketing in the school environment, more than half of the regulations did not mention any restriction. However, regulating advertising and marketing of food in the school environment is critical since childhood and adolescence are cycles of life in which there is a process of biological, cognitive, and emotional development. Therefore, from a legal point of view, these individuals have the right to complete protection and care [34, 35]. In this sense, children are not able to understand advertising messages in a context with which food is associated, nor can they distinguish the persuasive nature of these messages [36, 37].

Thus, because it is an audience still vulnerable to Marketing Communication actions and constantly exposed to advertising and food marketing content, regulatory measures must mention and describe ways to support a safe school environment. In addition, the Marketing Communication of ultra-processed foods can be associated with excessive consumption of these foods and, consequently, negatively impact the health and eating habits of exposed children and adolescents [37].

Regarding the points of excellence, supervision and social control regarding compliance with regulatory measures and the participation and monitoring exercised by society [38] in the school environment are very important. This aspect was mentioned in only 30.36% of the regulatory measures analyzed. Notably, the greater the participation of society in the fulfillment and strengthening of public policies that enable the promotion of healthy spaces, the greater the benefit to the community [39].

Regarding the scope of regulatory measures concerning school networks, more than half of the regulatory measures evaluated covered public and private schools. Notably, among the regulatory measures assessed in this study, 14 of them do not include private schools. This observation holds significance given that, according to the 2019 School Census [10], all the states and cities considered in this study, which possess regulatory measures, have a presence of private schools. This information sheds light on a critical gap in the regulatory framework, leaving a substantial portion of the school population, particularly in private educational institutions, needing adequate coverage and oversight concerning healthy food provisions. Addressing this disparity is crucial to ensure a comprehensive and equitable approach to promoting healthy dietary practices across all types of schools.

The situation in public schools raises concerns about the prevalence of obesogenic environments in private schools. Carmo et al. (2018) [40] conducted a study evaluating the food environment of schools participating in the Brazilian Study of Cardiovascular Risks in Adolescents (Estudo de Riscos Cardiovasculares em Adolescentes, ERICA) in 2013 and 2014. Their findings revealed that private schools tend to foster obesogenic environments, promoting the consumption of ultra-processed foods. Conversely, public schools were found to have a relatively healthier food environment.

It is essential to highlight that the PNAE has implemented various resolutions and guidelines to promote sustainable and healthy dietary practices within schools. This initiative is crucial for safeguarding the food environment, especially for students in state and city schools across Brazil [41, 42].

Furthermore, research has shown that students benefiting from PNAE experience advantages such as reduced consumption of ultra-processed foods [43] and lower incidences of obesity and hypertension [44]. Given this, it is imperative to direct special attention towards private schools when formulating regulatory measures, as they fall outside the scope of PNAE. Private schools’ more obesogenic food environment contributes to unhealthy dietary habits among children and adolescents [45–47], necessitating targeted strategies to address this disparity.

We considered the existence of laws, regulated using a decree, as a point of excellence. Although more than 80% of the sets of regulatory measures analyzed included laws, only three were regulated by a decree. In this regard, it is important to note that although a decree is subordinate to a law, since many laws are not self-applicable, they need regulations to be effective [25, 48]. Thus, when we have decrees regulating laws, the strength of these legal provisions makes it possible to implement them in schools and makes it difficult to repeal them when governments change.

When considering banning ultra-processed foods, only one regulatory measure cited “ultra-processed food” in the text. This lack of mention was considered regarding the measures published before the Dietary Guidelines for the Brazilian Population. However, about 30% of the measures were published after 2014, showing the need for constant improvement of the measures considering the advancement of discussions related to official dietary guidelines in Brazil [32].

The set of regulatory measures by the federal entity that showed the best score was from the Federal District, which included Law Number 5,146 of 2013, regulated by Decrees Number 36,900 of 2015 and Number 37,346 of 2016; Law Number 5,232 of 2013; and Law Number 6,475 of 2020. Despite not clearly mentioning ultra-processed foods, these laws and decrees restrict the marketing of unhealthy foods, meet all the other scoring criteria proposed in the score, and extend its scope to 50 m around schools.

It is important to point out that formulating and implementing regulatory measures concerning the food supply, marketing, and advertising in schools presents various challenges [20]. These obstacles often stem from industry resistance, critiquing regulatory design, and framing policies negatively in public discourse [49–51]. Consequently, judicial decisions may be constrained by differing interpretations of the provisions and objectives within these regulatory measures [52]. Despite these challenges, it is imperative to continually work on refining and strengthening the implementation of regulatory measures through actions and strategic evaluations, maintenance, and necessary adjustments tailored to specific contexts [53]. Considering this, involving the entire school community, including parents or guardians, school administrators, canteen staff, and civil society members, is vital for fostering consensus and promoting the effective formulation and application of regulatory measures [20, 53].

The present study has limitations: the search may not have included all regulatory measures in Brazil. The need for an official basis for consultation of existing and current Brazilian legal provisions makes the process vulnerable. However, we did our best to minimize this issue by searching the government websites and contacting them by e-mail. This study’s strength and innovative character is the creation of a score to evaluate the effectiveness of Brazilian regulatory measures in promoting healthier school food environments.

## Conclusions

The study’s findings underscore a significant gap in the current regulatory measures, indicating their inadequate promotion of wholesome and nutritious food within school premises. Notably, various Brazilian states and cities have initiated efforts to establish regulatory frameworks to foster a healthy food environment within schools. However, a critical need exists to align these measures with the latest recommendations outlined in the Food Guide for the Brazilian Population, specifically focusing on promoting healthy food environments.

A nationwide initiative led by the Ministry of Health and Education is imperative to bridge this gap effectively. This initiative should support states and cities in comprehensively revising and applying their legal provisions. It is important to note that the Model Law Project produced by Idec is an important support tool. Equally vital is the need to conduct targeted training programs for educators and other stakeholders to improve their understanding and proficiency in promoting a healthy school environment and act as enforcers of regulatory measures. By doing so, we can effectively promote healthy eating practices and healthy food environments in educational institutions.

### Electronic supplementary material

Below is the link to the electronic supplementary material.

Supplementary Material 1

## Data Availability

All data generated or analyzed during this study are included in this published article Supplementary Material 1.
