# Supplementary material for: Do Brazilian regulatory measures promote sustainable and healthy eating in the school food environment?
Source: BMC Public Health. 2023 Nov 6;23:2166. doi: 10.1186/s12889-023-17111-7 (PMC10626708; doi:10.1186/s12889-023-17111-7)
Supplement: Supplementary file 1 — Supplementary Material 1 [file 12889_2023_17111_MOESM1_ESM.docx]

**SUPPLEMENTARY MATERIAL**

**Supplementary Material 1.** Describes the regulatory measures evaluated in the study, organized by a federal entity.

| **Cities/States** | **Regulatory Measures** | **Link to Acess** | **Year of publication** | **Scope** | **Current Status** |
| --- | --- | --- | --- | --- | --- |
| **STATES** | | | | | |
| Acre | Law nº 3,134 | <https://encurtador.com.br/irzNT> | 2016 | Public and private | In force |
| Amazonas | Law nº 4,352 | https://www.legisweb.com.br/legislacao/?id=325875 | 2016 | Public and private | Repealed |
| Bahia | Ordinary Law nº 14,045 | <https://leisestaduais.com.br/ba/lei-ordinaria-n-14045-2018-bahia-altera-a-lei-no-13-582-2016-para-dispor-sobre-a-publicidade-infantil-nos-estabelecimentos-de-educacao-basica-no-estado-da-bahia> | 2018 | Public and private | In force |
|  | Law nº 13,582 | <https://goo.gl/4iPzTD> | 2016 | Public and private | In force |
| Ceará | Law n° 15,205 | <https://belt.al.ce.gov.br/index.php/legislacao-do-ceara/organizacao-tematica/educacao/item/1858-lei-n-15-205-de-19-07-12-d-o-24-07-12> | 2012 | Public | In force |
| Distrito Federal | Law nº 6,475 | <http://www.sinj.df.gov.br/sinj/Norma/2760e4f16c8b4887976bc86b7076adf6/Lei_6475_2020.html> | 2020 | Public | In force |
|  | Law nº 5,232 | <http://www.sinj.df.gov.br/sinj/Norma/75931/Lei_5232.html> | 2013 | Public | In force |
|  | Law nº 5,146 | <https://goo.gl/7Y9ZiB> | 2013 | Public and private | In force |
|  | Decree nº 36,900 | <https://goo.gl/UFjNgr> | 2015 | Public and private | In force |
|  | Decree nº 37,346 | <http://www.sinj.df.gov.br/sinj/Norma/14d039923cf848ac920cd005ea29954d/Decreto_37346_17_05_2016.html> | 2016 | Public and private | In force |
|  | Law nº 3,695 | <https://www.sinj.df.gov.br/sinj/Norma/51622/Lei_3695_08_11_2005.html> | 2005 | Public and private | Repealed |
| Espírito Santo | Ordinance nº 038R | <http://sinepe-es.org.br/?37/noticia/portaria-no-038-r> | 2010 | Public | In force |
|  | Ordinance nº 066 R | <https://document.onl/documents/normas-funcionamento-cantina-19-10-2015.html> | 2014 | Public and private | In force |
| Maranhão | Law nº 10,342 | <https://www.legisweb.com.br/legislacao/?id=305086> | 2015 | Public and private | In force |
|  | Law n° 11,196 | <https://www.legisweb.com.br/legislacao/?id=387976> | 2019 | Public and private | In force |
| Mato Grosso | Law nº 8,681 | <https://encurtador.com.br/cdW49> | 2007 | Public and private | In force |
|  | Law nº 8,944 | <https://encurtador.com.br/lozM8> | 2008 | Public and private | In force |
| Mato Grosso do Sul | Law nº 4,320 | <https://goo.gl/cYy7nS> | 2013 | Public | In force |
| Minas Gerais | Law nº15,072 | <https://www.almg.gov.br/consulte/legislacao/completa/completa.html?tipo=LEI&num=15072&ano=2004> | 2004 | Public and private | In force |
|  | Law nº 18,372 | <https://www.almg.gov.br/consulte/legislacao/completa/completa.html?num=18372&ano=2009&tipo=LEI> | 2009 | Public and private | In force |
|  | Resolution nº1,511 | <http://www.contagem.mg.gov.br/estudacontagem/wp-content/uploads/2017/05/1511-10-r.pdf> | 2010 | Public and private | In force |
|  | Decree nº 47,557 | <https://www.almg.gov.br/legislacao-mineira/DEC/47557/2018/> | 2018 | Public and private | Repealed |
| Paraíba | Law nº 10,431 | <https://goo.gl/U1CFHb> | 2015 | Public and private | In force |
| Paraná | Law nº 14,423 | <https://goo.gl/mJybMS> | 2004 | Public and private | In force |
|  | Law nº 16,085 | <https://encurtador.com.br/nrDTV> | 2009 | Private | In force |
|  | Law nº 14,855 | <https://www.legislacao.pr.gov.br/legislacao/pesquisarAto.do?action=exibir&codAto=6351&indice=1&totalRegistros=1> | 2005 | Public and private | In force |
| Piauí | Law nº 7,028 | <https://www.normasbrasil.com.br/norma/lei-7028-2017-pi_348142.html> | 2017 | Public and private | In force |
|  | Normative Instruction GSE/ADM nº 005 | <https://encurtador.com.br/wxXZ7> | 2018 | Public and private | In force |
| Rio de Janeiro | Law nº 7,394 | <https://www.legisweb.com.br/legislacao/?id=326291> | 2016 | Public and private | In force |
|  | Law nº 6,590 | <https://www.normasbrasil.com.br/norma/lei-6590-2013-rj_261873.html> | 2013 | Private | In force |
|  | Law nº 4,508 | <https://gov-rj.jusbrasil.com.br/legislacao/88467/lei-4508-05> | 2005 | Public and private | In force |
| Rio Grande do Norte | Law nº 9,434 | <https://www.normasbrasil.com.br/norma/lei-9434-2010-rn_152728.html> | 2010 | Public | In force |
| Rio Grande do Sul | Law nº 15,216 | <https://www.normasbrasil.com.br/norma/lei-15216-2018-rs_365652.html> | 2018 | Public and private | In force |
|  | Decree nº 54,994 | <https://encurtador.com.br/qCQ68> | 2020 | Public and private | In force |
|  | Law nº 13,027 | <https://www.normasbrasil.com.br/norma/lei-13027-2008-rs_154622.html> | 2008 | Public and private | In force |
| Rondônia | Ordinance nº1,851 | <https://www.jusbrasil.com.br/diarios/41177944/doero-03-10-2012-pg-27> | 2012 | Public | In force |
|  | Ordinance nº1,538 | <https://www.normasbrasil.com.br/norma/portaria-1538-2016-ro_320133.html> | 2016 | Public | In force |
| Roraima | Resolution nº 1 | <https://www.normasbrasil.com.br/norma/resolucao-1-2012-rr_243216.html> | 2012 | Public | In force |
| Santa Catarina | Law nº 12,061 | <https://goo.gl/pa8N9L> | 2001 | Public and private | In force |
| São Paulo | Joint Ordinance COGSP/CEI/DSE | <http://siau.edunet.sp.gov.br/ItemLise/arquivos/notas/portconj_cogsp_cei_dse(doe230305).htm> | 2005 | Public | In force |
|  | Law nº 17,340 | <https://www.al.sp.gov.br/repositorio/legislacao/lei/2021/lei-17340-11.03.2021.html> | 2021 | Public and private | In force |
| Sergipe | Law n° 8,178-A | <https://www.legisweb.com.br/legislacao/?id=337364> | 2016 | Public and private | In force |
| **cities** | | | | | |
| Aracaju/SE | Law nº 3,814 | <https://goo.gl/s52FnQ> | 2010 | Public and private | In force |
| Belo Horizonte/MG | Law nº 8,650 | <https://goo.gl/9dDwJU> | 2003 | Public and private | In force |
| Campo Grande/MS | Law nº 4,992 | <https://www.normasbrasil.com.br/norma/lei-4992-2011-campo-grande_172949.html> | 2011 | Public and private | In force |
| Cuiabá/MT | Law nº 4,382 | <https://cm-cuiaba-mt.jusbrasil.com.br/legislacao/573739/lei-4382-03> | 2003 | Public and private | In force |
|  | Law nº 4,589 | <https://cm-cuiaba-mt.jusbrasil.com.br/legislacao/571864/lei-4589-04> | 2004 | Public and private | In force |
| Curitiba/PR | Law nº 10,950 | <https://cm-curitiba.jusbrasil.com.br/legislacao/339198/lei-10950-04> | 2004 | Public and private | In force |
| Divinópolis/MG | Ordinary Law n° 7,163 | <https://www.divinopolis.mg.leg.br/leis/copy_of_legislacao-municipal> | 2010 | Public and private | In force |
| Florianópolis/SC | Law nº 5,853 | <https://goo.gl/b4KCDc> | 2001 | Public and private | In force |
| Fortaleza/CE | Law nº 8,824 | <https://encurtador.com.br/pxyRV> | 2004 | Public and private | In force |
| Itapetininga/SP | Law nº 5,320 | <https://legislacaodigital.com.br/Itapetininga-SP/LeisOrdinarias/5320> | 2009 | Public and private | In force |
| Jataí/GO | Ordinary Law nº 3,230 | <https://www.jatai.go.leg.br/ta/3635/text>? | 2011 | Public | In force |
| Juiz de Fora/MG | Law n° 12,121 | <https://jflegis.pjf.mg.gov.br/norma.php?chave=0000033092> | 2010 | Public and private | In force |
| Manaus/AM | Law nº 1,414 | <https://goo.gl/9W56pg> | 2010 | Public and private | In force |
|  | Decree nº 741 | <https://www.legisweb.com.br/legislacao/?id=175982> | 2011 | Public and private | In force |
| Palmas/TO | Law nº 1,210 | <https://goo.gl/YKcoZA> | 2003 | Public | In force |
| Palmitinho/RS | Law nº 2,628 | <https://palmitinho.cespro.com.br/visualizarDiploma.php?cdMunicipio=7725&cdDiploma=20172628&NroLei=2.628&Word=&Word2=> | 2017 | Public and private | In force |
| Pelotas/RS | Municipal Law nº 5,778 | <https://encurtador.com.br/kBJS2> | 2011 | Public and private | In force |
| Petrolina/PE | Municipal Law nº 2,436 | <https://encurtador.com.br/qxDY2> | 2011 | Public and private | In force |
| Porto Alegre/RS | Law nº 10,167 | <https://goo.gl/euy92Q> | 2007 | Public and private | In force |
| Ribeirão Preto/SP | Resolution nº 16 | <https://www.ribeiraopreto.sp.gov.br/portal/pdf/educacao296202111.pdf> | 2002 | Public | In force |
| Rio de Janeiro/RJ | Decree nº 21,217 | <http://www.rio.rj.gov.br/dlstatic/10112/5118607/4132721/Decreton21.217de1.deabrilde2002.pdf> | 2002 | Public | In force |
|  | Ordinance nº02 | <http://189.28.128.100/nutricao/docs/geral/regula_comerc_alim_escolas_exper_estaduais_municipais.pdf> | 2004 | Public and private | Repealed |
| Salvador/BA | Law nº 8,292 | <https://goo.gl/NDvF6T> | 2012 | Public and private | In force |
| Santos/SP | Law n°2,327 | <https://cm-santos.jusbrasil.com.br/legislacao/517556/lei-2327-05> | 2005 | Public | In force |
| Teresina/PI | Law nº 5,380 | <https://www.normasbrasil.com.br/norma/lei-5380-2019-teresina_378612.html> | 2019 | Public and private | In force |
| Vitória/ES | Law nº 8,106 | <https://www.legisweb.com.br/legislacao/?id=127267> | 2011 | Public and private | In force |
|  | Law nº 6,786 | <https://goo.gl/abZKSd> | 2006 | Public | In force |

**Supplementary Material 2.** Manual of application of the framework to evaluate the measures in the federal entities.

**GUIDELINES FOR THE APPLICATION OF THE REGULATORY MEASURES ASSESSMENT FRAMEWORK**

**1**.    **PRESENTATION**

In 2018, the Brazilian Institute of Consumer Protection (IDEC) produced the document “Healthy Eating in Schools: Guide for Cities,” aimed at public managers and technical teams in the areas of health and education to promote healthy school food environments as one of the actions to confront obesity in childhood and adolescence. One of the measures recommended in the Guide is the development of effective legal provisions to assist school managers in promoting healthy school food environments. As a tool to assist municipal managers in developing these legal provisions, a Model Law Project was presented with the necessary information to create effective measures aimed at the school food environment.

The framework proposed by the Model Law Project aims to evaluate existing and current regulatory measures aimed at the school food environment in Brazilian states and cities based on a sum of points. This framework is based on seven domains constituting the school food environment: 1. Food and Nutritional Education; 2. Distribution and Marketing of Food; 3. Marketing Communication; 4. Supervision of the Implementation of the Regulatory Measure; 5. Scope of the Regulatory Measure; 6. Regulatory Power of the Regulatory Measure 7. Mention of Ultra-processed Foods.

The framework for assessing regulatory measures is essential for identifying which regulatory measures do not include all the domains that influence the diet of children and adolescents in the school environment, as well as being the basis for reformulating such regulatory measures. In addition, such a framework can support states and cities in building healthier school food environments.

Implementing regulatory measures that include all the multiple dimensions that influence school children's diet is essential for promoting a healthy environment in public and private schools (BRASIL, 2007; REED, 2014; COHEN, 2015; MICHA, 2018). In Brazil, there is no tool for evaluating regulatory measures on the school food environment, which can hinder the understanding, supervision, and implementation of such measures and the promotion of sustainable, adequate, and healthy eating in the school environment.

**CONSTRUCTION OF THE REGULATORY MEASURES EVALUATION FRAMEWORK**

The Model Law Project (<https://idec.org.br/projeto-de-lei-para-escolas>) is based on seven domains that constitute the school food environment. They were based on a sum of points and used to build the score to evaluate the existing and current regulatory measures aimed at the school food environment in Brazilian states and cities. The domains comprise: 1. Food and Nutritional Education; 2. Distribution and Marketing of Food; 3. Marketing Communication; 4. Supervision of the Implementation of the Regulatory Measure; 5. Scope of the Regulatory Measure; 6. Regulatory Force of the Regulatory Measure 7. Mention of Ultra-processed Foods. The last one is supported by the dietary guidelines issued by the Dietary Guide for the Brazilian Population, based on the NOVA classification, in which foods are categorized according to the extent and degree of processing as unprocessed or minimally processed foods, processed foods, processed culinary ingredients and ultra-processed foods (Monteiro et al., 2019).

A framework based on the Model Law Project was proposed to evaluate the regulatory measures aimed at the school food environment. Based on a sum of points, the score seeks to evaluate existing and current regulatory measures in Brazilian states and cities. The classification based on the score allowed the measures to be grouped into three categories according to the number of points received:

| **0 to 3 points**  Regulatory measures exist and need to be improved to fulfill their function of promoting sustainable, adequate, and healthy eating in the school food environment  **4 to 7 points**  Regulatory measures partially perform their function of promoting sustainable, adequate, and healthy eating in the school food environment  **8 to 12 points**  Regulatory measures fulfill their function of promoting sustainable, adequate and healthy eating in the school food environment |
| --- |

**APPLICATION OF THE FRAMEWORK**

The application of the framework can be evaluated in two ways: by regulatory measure or by the set of regulatory measures existing in the state and/or city. When the application is carried out on a set of measures, if any measure of this set presented one of the items evaluated, the entire group received the score. This joint assessment is justified by the complementary nature of the regulatory measures in force in the state and/or city regulatory measures in force. When a city or state has more than one regulatory measure (two laws and one decree, for example), this set is evaluated and considered when the framework is applied.

In constructing the framework, the domains described below were considered, and, in each domain, examples of regulations that reached total scores are presented.

**● Food and Nutritional Education**

**0 (zero) point: Not mentioned in the regulatory measure**

The regulation does not receive a score when it does not mention any Food and Nutrition Education action.

**1 (one) point: It is mentioned in the regulatory measure, without provisions for its development**

Featured: Minas Gerais - SEE Resolution No. 1.511, of February 26, 2010

Art. 1 The food program in the state schools of Minas Gerais must observe the determinations of RESOLUTION/CD/FNDE No. 38, of July 16, 2009 – of the National Fund for the Development of Education of the Ministry of Education – and the guidelines of the booklets "Menu Suggestions" and "Canteen Manual," edited by the State Department of Education of Minas Gerais, aiming at healthy and sustainable food, the use of varied and safe foods, the correct preparation of foods and the promotion of good eating habits of the students of Minas Gerais state schools.

Sole paragraph. The school should develop activities that reinforce healthy eating habits.

**2 (two) points: It is mentioned in the regulatory measure and provides for its development**

Featured: Rio Grande do Sul - Decree No.5 4,994 of January 17, 2020

Art. 10. Schools may carry out educational campaigns and actions, including a transversal pedagogical approach, on the topics listed below:

I – Food and culture;

II – Balanced meal, food groups, and their functions;

III – Food and media;

IV – Healthy habits and lifestyles;

V – Fruits and vegetables: preparation, consumption and their importance for health;

VI – Hunger and food safety; and

VII – Scientific data on the harms of food consumption whose marketing is prohibited by Law No. 15.216/2018 and by this Decree

Art. 11. The Department of Health and the Department of Education will promote educational actions and will organize informational material on the content of this Decree, including guidance on healthy eating habits in the school environment.

**● Distribution and Marketing of Food**

**0 (zero) point:** **There is no mention of any type of regulation of the distribution and marketing of food in the school environment in the regulatory measure**

The regulatory measure does not receive a score when it does not mention any regulation aimed at the marketing of food in the school environment, such as the prohibition of unhealthy food and food allowed for marketing in and around the schools.

**1 (one) point:** **The regulation of the distribution and marketing of food in the school environment is mentioned in the regulatory measure, without distinguishing which foods are prohibited or allowed**

Featured: Paraná - Law No. 14,423 of June 2, 2004

**Art. 2.** Given the nutritional precept and following the previous article, it is expressly forbidden, in snack and beverage offerings or similar, to sell the following:

**a)** any alcoholic beverages;

**b)** candies, lollipops and chewing gums;

**c)** soft drinks and artificial juices;

**d)** processed snacks;

**e)** fried snacks; and

**f)** processed popcorn.

**§ 1.** The food establishment should make two kinds of seasonal fruits available to students, aiming at the choice and nutritional enrichment of the students.

**§ 2.** The sale of foods and soft drinks containing, in their chemical compositions, nutrients that are proven to be harmful to health is prohibited.

**2 (two) points: The regulation of the distribution and marketing of food in the school environment is mentioned in the regulatory measure, distinguishing which foods are prohibited or allowed**

Featured: Pelotas/RS - Municipal Law No. 5.778 of January 21, 2011

Art. 3 The administration of the School Canteen should receive guidance on nutrition and healthy snacks from the nutritionists of the Municipal Public Network.

Art. 4 The marketing of the products listed below is prohibited in the environment of early childhood education, primary and secondary education schools of public and private education networks in the city of Pelotas:

I - Candies, lollipops, chewing gums, filled cookies;

II - Soft drinks and artificial juices;

III - Processed snacks;

IV - Fried food in general;

V - Processed popcorn;

VI - Foods whose preparation uses hydrogenated vegetable fat.

Sole Paragraph - The prohibition referred to in this article extends to street vendors near schools.

Art. 5 The school canteen will offer daily at least one variety of unprocessed seasonal fruit, whole or in pieces, or in the form of juice, preferably with ingredients produced in the region of Pelotas.

Art. 6 The contract between the school and the school canteen, when applicable, will contain clauses observing this law.

Sole Paragraph - In public tenders, the draft contract that is part of the respective notice for the operation of school canteen services will contain clauses specifying marketable items in compliance with the provisions of this Law.

***●* Marketing Communication**

**0 (zero) point: There is no mention of any type of regulation of marketing communication in the school environment in the regulatory measure**

The regulatory measure does not receive a score when it does not mention any regulation of marketing communication in the school environment, such as encouraging the consumption of unhealthy foods, sponsorship of school programs and activities by companies; advertising campaigns in the school space; distribution of commercial samples in the school space; sponsorship of pedagogical material by companies.

**1 (one) point: the regulatory measure prohibits marketing communication in the school environment**

Featured: Rio de Janeiro - Law No. 4508, of January 11, 2005

Art. 1 - It is forbidden to sell, purchase, manufacture, and distribute products that contribute to childhood obesity in bars, canteens, and the like located in public and private schools located in the state of Rio de Janeiro.

Art. 2 - The following products are included in the provisions of the "heading" of Article 1: snacks, candies, chocolates, gum-based sweets, chewing gum, lollipops, caramels, processed powdered refreshment, soft drinks, any food handled at school or in a non-accredited environment for making food preparation, alcoholic beverages, foods with more than 3 (three) grams of fat in 100 (one hundred) kcal of the product, with more than 160 (one hundred and sixty) mg of sodium in 100 (one hundred) kcal of the product and foods containing artificial colors, preservatives or anti-oxidants (listed on the nutritional labeling available on the packaging), foods without labeling, nutritional composition and expiration date.

Sole Paragraph - It is also forbidden to advertise any products listed in Article 2 on school premises.

**2 (two) points:** **The marketing of the prohibited foods in the school environment is prohibited by the regulatory measure, and describes the prohibited resources**

Featured: Campo Grande/MS - Law No. 4.992 of September 30, 2011

Art. 7 The marketing of the following products is prohibited:

I - Candies, lollipops, chewing gums, filled cookies;

II - Soft drinks and artificial juices;

III - Processed snacks;

IV - Fried food in general;

V - Processed popcorn;

VI - Alcoholic drinks;

VII - Processed foods whose percentage of calories from saturated fat exceeds 10% (ten percent) of the total calories;

VIII - Foods whose preparation uses hydrogenated vegetable fat.

Art. 11 Advertising of products whose marketing is prohibited by this law is prohibited in the school environment.

Sole paragraph. The prohibition contained in this article extends to advertising modalities through sponsorship of school activities, including extracurricular activities.

*●*  **Supervision of the Implementation of the Regulatory Measure**

**1 (one) point: The regulatory measure provides for supervision and social control (by health surveillance, consumer protection agencies, parent-teacher association or education agency)**

Featured: Federal District - Decree No. 36.900 of November 23, 2015

Art. 9 The State Department of Health of the Federal District is responsible for the supervision and sanitary control of canteens located in the units of the education network, as provided for in District Law No. 5.321, of March 6, 2014.

***●*  Scope of the Regulatory Measure**

**1 (one) point: The regulatory measure covers private schools**

Featured: Mato Grosso - Law No. 8.681 of July 13, 2007

Art. 1 Foods supplied or made available in the canteens of school units, public and private, in the State of Mato Grosso that serve early childhood and basic education must observe the standards of nutritional quality and life indispensable to the health of students.

***●*  “Power” to Regulate the Regulatory Measure**

**1 (one) point: The regulatory measure is a law**

Featured: Ceará - Law No. 15.205, of July 19, 2012

**2 (two) points: The regulatory measure is a law and is regulated by a decree**

Featured: Manaus/AM - Law No. 1.414 of January 22, 2010 regulated by Decree No. 741 of January 12, 2011

***●* Mention of Ultra-processed Foods**

**1 (one) point:** **The regulatory measure prohibits ultra-processed foods**

Featured: Maranhão - Law No. 11.196 of December 19, 2019

Art. 1 This law establishes general rules and basic criteria for the promotion of healthy eating and determines the exclusion of ultra-processed and sugary foods in public and private schools within the scope of the State of Maranhão.

Art. 2 For healthy eating, the following are considered ultra-processed and sugary foods:

I - Healthy eating is based on balance and variety in intake, comprising proteins, fats, carbohydrates (including fiber), vitamins, and minerals.

II - Ultra-processed and sugary foods are manufactured with several steps, processing techniques, and ingredients, many exclusively for industrial use.
